# Introduction to this issue of Perspectives on Medical Education

**DOI:** 10.1007/s40037-013-0077-9

**Published:** 2013-09-14

The Netherlands Association of Medical Education has 14 working groups (special interest groups) dealing with different aspects of medical education. Some of these groups have a long-standing history, other groups started only recently as a response to new developments in the field of medical education. The Editorial Board of Perspectives on Medical Education (PME) has invited all working groups to make a proposal for a special issue devoted to the working group’s interest. The Board’s intention is to publish a special issue once or twice a year.

We are very happy that several working groups have responded positively and are now preparing proposals for a special issue of PME. This is the first special issue of PME. It was composed with the help and initiatives of the working group on ‘scientific education in undergraduate medical programmes’. Some manuscripts have been written by members of the working group, others have been written by authors following an invitation. During the preparation of this issue the chair of the working group, Professor Friedo Dekker, has acted as guest editor. He worked very closely with Dr. Renée Stalmeijer, associate editor of PME, who coordinated the composition of the issue and the review process of the manuscripts. We thank the members of the working group and all other authors for their contributions. We hope that this special issue is of interest to our readers and will inspire them to further research and submitting new manuscripts to PME.

Editorial Board of PME

